# Factors associated with HCV risk practices in methadone-maintained patients: the importance of considering the couple in prevention interventions

**DOI:** 10.1186/1747-597X-9-37

**Published:** 2014-09-10

**Authors:** Perrine Roux, Caroline Lions, Laurent Michel, Marion Mora, Jean-Pierre Daulouède, Fabienne Marcellin, Bruno Spire, Alain Morel, Patrizia M Carrieri

**Affiliations:** 1 INSERM U912 (SESSTIM), Marseille, France; 2 Université Aix Marseille, IRD, UMR-S912, Marseille, France; 3 ORS PACA, Observatoire Régional de la Santé Provence Alpes Côte d’Azur, Marseille, France; 4 INSERM, Research Unit 669, Paris, France; 5 Univ Paris-Sud and Univ Paris Descartes, UMR-S0669, Paris, France; 6 Centre Pierre Nicole, Paris, France; 7 Centre medico-social Bizia, Bayonne, France; 8 Oppelia, Paris, France; 9 INSERM U912 – ORS PACA, 23 rue Stanislas Torrents, 13006 Marseille, France

**Keywords:** Methadone, HCV transmission, Risk practices, Cocaine, Sharing, Couples

## Abstract

**Background:**

One important public health issue associated with opioid use today is the risk of hepatitis C (HCV) infection. Although methadone maintenance may help to decrease HCV-related risk practices, HCV risk behaviors persist and are strongly associated with specific substance use patterns, mental status and social context. The ANRS-Methaville study gave us the opportunity to better disentangle the different relationships between these various factors and HCV risk practices.

**Methods:**

The ANRS-Methaville multisite randomized trial was designed to assess the feasibility of initiating methadone in primary care by comparing it with methadone initiation in specialized centers. This study recruited 195 participants initiating methadone maintenance and followed up for 12 months. Longitudinal data from this trial was used to acquire a greater understanding of HCV risk practices and their pattern of correlates in this population. We selected 176 patients who had data on HCV risk practices at M0 and M12, accounting for 312 visits. HCV risk practices were defined as follows: sharing needles or syringes, sharing drug paraphernalia, getting a tattoo or having a piercing in a non-professional context, sharing toiletry items. To identify factors associated with HCV risk practices, we performed a mixed logistic regression analysis.

**Results:**

HCV risk practices were reported by 19% and 15% of participants at baseline and M12, respectively. After adjustment for age, cocaine use and alcohol dependence as well as suicidal risk, living in a couple with a non-drug user and in a couple with a drug user were both independent predictors of HCV risk practices (OR[CI95%] = 4.16 [1.42-12.12]; OR[CI95%] = 9.85 [3.13-31.06], respectively).

**Conclusions:**

Identifying individuals at risk of HCV transmission during methadone treatment such as stimulant users, alcohol dependent individuals, and those at suicidal risk is necessary to optimize response to treatment. Innovative prevention approaches tailored to couples are also urgently needed and could decrease HCV-risk in this population.

The trial is registered with the French Agency of Pharmaceutical Products (ANSM) under the number 2008-A0277-48, the European Union Drug Regulating Authorities Clinical Trials. Number Eudract 2008-001338-28, the ClinicalTrials.gov Identifier: NCT00657397 and the International Standard Randomised Controlled Trial Number Register ISRCTN31125511.

## Background

Methadone maintenance treatment (MMT) is recognized as the gold standard for effective treatment for opioid dependence [1] as it can lead to a major reduction in the use of [2] or total abstinence from illicit opioids [3]. Furthermore, MMT has other positive outcomes such as social rehabilitation, improvement in psychological and psychiatric status and reduction in risk behaviors associated with HIV and HCV transmission. In people who use drugs (PWUD) and especially in people who inject drugs (PWID), HCV infection prevention and re-infection after being cured of HCV remain major public health issues. Among PWID, the prevalence of HCV infection may be very high [4] and this is the result not only of the current lack of availability of a combined package of prompt preventive interventions at a national level [5] but also of individual risk factors. Indeed, this explains why HCV prevalence varies so greatly, and is independent of the prevalence of drug use. It is important to distinguish the different levels of risk according to the practices engaged in by individuals. Besides the sharing of syringes/needles which is known to be the main route of HCV transmission [6], the sharing of other injecting paraphernalia [7] is also considered a high risk route of HCV transmission. Other lower risk factors have also been identified, such as the sharing of straws in drug sniffing [8], getting tattoos or piercings in a non-professional context [9] and the sharing of personal toiletry items in a household context [10]. One study has shown that HCV-positive patients are unaware of the risk of certain routes of transmission, such as the sharing of personal items including toothbrushes, razors and nail scissors [11].

Although research has highlighted a reduction in injection practices thanks to MMT programs [12] in PWID, it is also acknowledged that such preventative interventions alone may not have a strong enough impact on reducing HCV incidence in drug users [13]. Accordingly, we used data from the ANRS Methaville trial to better document HCV risk practices in methadone patients and to investigate the influence of specific individual risk factors in order to suggest improvements in standard preventive interventions like MMT.

## Methods

### Study design

The ANRS-Methaville study is a multi-site, open-label, randomized, controlled, non-inferiority, pragmatic trial designed to assess the feasibility of initiating methadone in primary care by comparing methadone initiation in specialized centers with methadone initiation provided by primary care physicians. Between January 2009 and January 2010, 195 men and women were recruited in 10 sites randomly allocated to initiate methadone in specialized centers (standard care) or in primary care physicians’ offices. Individuals who had a triple dependence on opioids, alcohol and benzodiazepines were not included in the study. The full protocol is described elsewhere [14]. The study protocol was approved by the Ethics Committee of Persons Protection in Paris, France. All individuals provided written, informed consent before participating in the study.

At initiation, 3, 6 and 12 months (M0, M3, M6 and M12 respectively), a medical questionnaire was filled in by the physician. A self-reported questionnaire was completed by the patient during each medical visit. After each medical visit, a computer-assisted telephone interview (CATI) was conducted by trained, non-judgmental staff. The primary outcome was abstinence from street-opioids at 12 months (M12) (with an underlying 15% non-inferiority hypothesis for PC).

### Data collection

At enrollment, we collected the following information: 1) Socio-demographic characteristics: gender, age, educational level, children, employment status, housing (owner or renter, living in family home, in a hospital or clinic, in a social care institution/hostel, in a friend’s home, in a hotel room, no home, other); 2) History of drug use: history of drug overdose, of drug injection, age at first regular drug use and at first injection. Unstable housing was defined as not being a home owner or renter. Employment status, housing and living in a couple were evaluated again at M12.

Alcohol consumption was assessed at M0 and M12 using the Alcohol Use Disorders Test (AUDIT) with a cut-off point ≥ 13 being used to identify alcohol dependence [15].

At each follow-up visit, drug use and drug injection in the previous month were assessed using the Opiate Treatment Index (OTI) [16]. Individuals who reported using cocaine or having injected drugs at least once in the previous month were defined as cocaine users or current injectors, respectively. The patient’s social network was assessed at enrollment and M12, by asking participants two questions, as follows: how much of the time they lived with someone who consumed heroin in the previous 6 months (dichotomized as follows: from “all of the time” to “less than half of the time” vs. “never”) and how many of the people who they were in regular contact with had used drugs in the previous 6 months (dichotomized as follows: from “all” to “less than half” vs. “none”).

We combined the two variables “living in a couple” and “living with someone who consumed heroin” and obtained four categories variables: “living alone”; “living in a couple with a non-drug user”; “living with a drug user not in a couple”, “living in a couple with a drug user”.

The prevalence of HCV risk practices was also documented using a set of questions extracted from the Blood-Borne Virus Transmission Risk Assessment Questionnaire (BBV-TRAQ) [17], which is a standardized questionnaire specifically adapted for this purpose, and also from the questionnaire used by Lucidarme et al. in their longitudinal study [18]. HCV risk practices in the previous month were assessed using a question about the sharing of injecting paraphernalia (lending and borrowing), needle sharing (lending and borrowing) and straw sharing. We also evaluated HCV risk practices related to other skin penetration practices (sharing of toiletry items, tattoos/piercings in a non-professional context and blood contact) in the previous month using the BBV-TRAQ questionnaire. We built a variable called “HCV risk practices”, which served as the study outcome measure, and was defined as reporting at least one of these HCV risk practices during the previous month.

### Self-reported and medical questionnaires

Depressive symptoms and suicidal risk were assessed at baseline and M12. For the former the Center for Epidemiological Study Depression scale (CES-D) was used during the phone interview. This enabled us to the compute a global depression score ranging from 0 to 60, with gender-specific cut-off values (23 for women and 17 for men). For the latter, we used the Beck hopelessness scale (BHS) which formed part of the patient self-administered questionnaire. A cut-off score of nine on the BHS is predictive of possible suicide risk behavior [19].

HCV testing was performed under the principles of routine clinical practice and was not something specific to the trial. At each medical visit participants were asked for the results of their most recent HCV test (positive, negative or unknown if the patient could not remember or if no test had ever been performed).

### Statistical analysis

We used a chi-squared test and a Wilcoxon test for, respectively, categorical and continuous variables, in order to compare characteristics of patients at enrollment in terms of HCV risk practices. The impact of time on methadone treatment and potential risk factors on HCV risk practices was assessed using a Logistic mixed model. We tested whether the following factors were associated with reporting at least one HCV risk practice: 1) socio-demographic characteristics: sex, age, high school certificate, having children, employment, living in a couple, unstable housing; 2) Alcohol consumption and drug consumption other than opiates : alcohol dependence, cocaine use, current injection; 3) Mental health problems: suicidal risk and depressive symptoms; 4) History of drug use: history of drug overdose and of drug injection, age at first regular drug use and at first injection; 5) Drug using network: drug using friends and living in a couple (or not) with a drug user (or not).

All variables associated with the outcome with a p-value lower than 0.25 in univariate analysis were considered eligible for the multivariate model. A stepwise procedure was used to identify the best model, and variables were removed one at a time based on a P-value of >0.05.

In addition, we performed a sensitivity analysis excluding the sharing of toiletry items from the outcome HCV risk practices. To verify which correlates still remained associated with this outcome, we used a Poisson regression model.

## Results and discussion

### Sample description

Table 1 describes the sample at baseline. Among the 176 individuals enrolled in the study and who had available data about HCV risk practices, 15% were female and the median (IQR) age was 32 (27-38) years. More than one third (35%) had a high school certificate and 37% had children. At baseline, half of the sample was employed and almost two-thirds had unstable housing. Forty percent were alcohol dependent, 28% reported using cocaine during the previous month and 15% reported current injecting drug use. The median (IQR) age at first regular drug use was 20 (18-24) years. Almost one-third of the sample was at risk of suicide and 39% had depressive symptoms. With respect to living alone or not and partner status, 55% reported living alone, 19% were living in a couple but not with a drug user, 14% were living with a drug user but not in a couple and 12% were living in a couple with a drug user. At baseline, 3 (2%) individuals reported being HIV positive, 26 (18.31%) HCV positive and 15% did not know their HCV status.

**Table 1:** Baseline characteristics (n (%) or median (IQR) of the sample used for the analyses (n = 176)): ANRS-Methaville trial

	**At least one HCV risk practice**			
	**No**	**Yes**	**Total**	***P†***
	**n = 142 (80.7%)**	**n = 34 (19.3%)**		
**Gender**				
Male	128 (90.1)	21 (61.8)	149 (84.7)	*<10*^*-3*^
Female	14 (9.9)	13 (38.2)	27 (15.3)	
**Age**^§^	34 (27-39)	29 (26-33)	32 (27-38)	*0.02*
**French High School Certificate (Bac)**				
< Bac	95 (66.9)	20 (58.8)	115 (65.3)	*0.37*
≥ Bac	47 (33.1)	14 (41.2)	61 (34.7)	
**Having child(ren)**				
No	87 (61.3)	23( 67.7)	110 (62.5)	*0.49*
Yes	55 (38.7)	11 (32.3)	66 (37.5)	
**Employment**				
No	66 (46.5)	20 (58.8)	86 (48.9)	*0.20*
Yes	76 (53.5)	14 (41.2)	90 (51.1)	
**Unstable housing**				
No	54 (38.0)	12 (35.3)	66 (37.5)	*0.77*
Yes	88 (62.0)	22 (64.7)	110 (62.5)	
**Alcohol dependence**^‡^				
No	122 (88.4)	26 (76.5)	148 (86.1)	*0.07*
Yes	16 (11.6)	8 (23.5)	24 (14.0)	
Cocaine consumption*				
No	101 (74.8)	20 (58.8)	121 (71.6)	*0.07*
Yes	34 (25.2)	14 (41.2)	48 (28.4)	
Current drug injection*				
No	111 (86.72)	26 (76.5)	137 (84.6)	*0.14*
Yes	17 (13.28)	8 (23.5)	25 (15.4)	
Suicidal risk**				
No	90 (74.4)	14 (50.0)	104 (69.8)	*0.01*
Yes	31 (25.6)	14 (50.0)	45 (30.2)	
Depressive symptoms***				
No	87 (64.0)	17 (50.0)	104 (61.2)	*0.14*
Yes	49 (36.0)	17 (50.0)	66 (38.8)	
**Age at first regular drug use**^§^	21 (18-25)	20 (17-21)	20 (18-24)	*0.02*
**Drug-using friends**				
No	42 (30.0)	7 (20.6)	49 (28.2)	*0.27*
Yes	98 (70.0)	27 (79.4)	125 (71.8)	
**Living**				
Alone	85 (61.2)	10 (29.4)	95 (54.9)	*0.002*
In a couple, not with a drug user	26 (18.7)	7 (20.6)	33 (19.1)	
With a drug user, not in a couple	15 (10.8)	9 (26.5)	24 (13.9)	
In a couple with a drug user	13 (9.4)	8 (23.5)	21 (12.1)	
**VHC status**				
Negative	84 (68.9)	22 (73.3)	106 (69.7)	*0.89*
Positive	19 (15.6)	4 (13.3)	23 (15.1)	
Unknown	19 (15.6)	4 (13.3)	23 (15.1)	

^†^Chi-squared test or Wilcoxon test; ^§^in years; ^‡^AUDIT score ≥13; *During the previous 4 weeks; **Beck ≥ 9; ***CES-D score >17 for males and >23 for females.

### HCV risk practices during the 12-month follow-up

Table 2 presents all the HCV risk practices at M0 and M12 using a univariate mixed model. At baseline, 11 (6%) individuals reported at least one HCV risk practice related to drug use (sharing of needles, injecting paraphernalia or straws) and 26 (16%) at least one HCV risk practice related to non-drug use practices (sharing of toiletry items, tattoos/piercings or blood contact). At baseline, 34 (19%) individuals reported at least one HCV risk practice (related or not to drug use).

**Table 2:** Proportion of the HCV risk practices at baseline and at M12 and impact of time on HCV risk practices: univariate mixed models

	**Baseline (MO)**	**M12**	**OR [95% CI]**	***p***
	**n (%)**	**n (%)**	**M12 vs. M0**	
**Sharing injection paraphernalia**	1 (0.6%)	1 (0.7%)	1.22 [0.08-19.59]	*0.89*
**Needle sharing**	3 (1.7%)	1 (0.7%)	0.40 [0.04-3.89]	*0.43*
**Straw-sharing**	9 (5.1%)	5 (3.5%)	0.64 [0.19-2.19]	*0.48*
**At least 1 HCV risk practice associated with drug use**	11 (6.3%)	6 (4.1)	0.63 [0.21-1.90]	*0.42*
**At least 1 HCV risk practice associated with other practices**	26 (16%)	18 (12%)	0.65 [0.30-1.45]	*0.29*
**At least 1 HCV risk practice**	34 (19%)	22 (15%)	0.68 [0.33-1.41]	*0.30*

### Factors associated with at least one HCV risk practice

Table 3 shows the factors associated with at least one HCV risk practice (related or not to drug use) in the univariate analysis. The following socio-demographic factors were associated with at least one HCV risk practice: female gender, being younger, having a high school certificate. With respect to drug and alcohol use, alcohol dependence, cocaine use and current drug injection were associated with the outcome. In addition, a longer history of regular drug use was linked to a higher risk of HCV risk practices. In terms of psychiatric comorbidities, individuals presenting depressive symptoms or suicidal risk were more likely to report HCV risk practices. Living in a couple was associated with more HCV risk practices. Compared with patients who were living alone, there was an increasing risk of HCV risk practices for those living in a couple with a non-drug user, for those not living in a couple but with a drug user and for those living in a couple and with a drug user. Furthermore, those who reported having drug-using friends were more likely to report HCV risk practices. HCV status was not associated with the outcome.

**Table 3:** **Factors associated with at least one HCV risk practice: univariate and multivariate**^**£**^**mixed models (n = 321 visits for 176 patients)**

	**Number of visits (%) or median (IQR)**	**Number of patients**	**OR**	**[95% CI]**	***p***	***aOR***	**[95% CI]**	***p***
**Gender**								
male	273 (85.1)	149	1					
female	48 (14.9)	27	6.24	[1.88-20.79]	*0.003*			
**Age**^§^	32 (27-38)		0.89	[0.83-0.96]	*0.004*	0.91	[0.85-0.97]	*0.01*
**High School Certificate**								
No	207 (64.5)	115	1					
Yes	114 (35.5)	61	2.48	[0.94-6.54]	*0.07*			
**Having child(ren)**								
No	202 (62.9)	110	1					
Yes	119 (37.1)	66	0.91	[0.35-2.41]	*0.85*			
**Employment**								
No	134 (41.7)	101	1					
Yes	187 (58.3)	124	0.99	[0.43-2.31]	*0.99*			
**Unstable housing**								
No	109 (34.0)	77	1					
Yes	212 (66.0)	129	0.71	[0.29-1.79]	*0.47*			
**Alcohol dependence** ‡								
No	279 (88.0)	158	1			1		
Yes	38 (12.0)	28	4.20	[1.23-14.32]	*0.02*	4.19	[1.53-11.47]	*0.01*
Cocaine use*								
No	245 (78.0)	149	1			1		
Yes	69 (22.0)	56	3.81	[1.51-9.62]	*0.01*	2.52	[1.05-6.04]	*0.04*
Current drug injection*								
No	274 (89.3)	156	1					
Yes	33 (10.8)	27	3.64	[1.07-12.48]	*0.04*			
Suicidal risk**								
No	189 (74.4)	121	1			1		
Yes	65 (25.6)	52	2.18	[0.92-5.17]	*0.08*	2.44	[0.99-6.02]	*0.05*
Depressive symptoms***								
No	210 (66.9)	132	1					
Yes	104 (33.1)	79	2.04	[0.84-4.96]	*0.12*			
**History of drug overdose**								
No	285 (88.8)	156	1					
Yes	36 (11.2)	20	0.65	[0.13-3.10]	*0.59*			
**History of drug injection**								
No	165 (51.7)	89	1					
Yes	154 (48.3)	86	1.64	[0.64-4.19]	*0.31*			
**Age at first regular drug use**^§^	20 (18-24)		0.84	[0.73-0.96]	*0.01*			
**Age at first injection**^§^	22 (19-26)		0.96	[0.84-1.10]	*0.55*			
**Drug using friends**								
No	114 (36.0)	81	1					
yes	203 (64.0)	134	3.03	[1.23-7.43]	*0.02*			
**Living**								
Alone	171 (53.9)	110	1			1		
In a couple, not with a drug user	78 (24.6)	56	2.65	[1.03-6.84]	*0.04*	4.16	[1.42-12.12]	*0.02*
With a drug user, not in a couple	36 (11.4)	32	4.78	[1.60-14.30]	*0.01*	2.46	[0.78-7.77]	*0.13*
In a couple with a drug user	32 (10.1)	27	7.95	[2.58-24.51]	*<10*^*-3*^	9.85	[3.13-31.06]	*<10-3*
**VHC status**								
Negative	195 (69.4)	106	1					
Positive	42 (15.0)	23	0.64	[0.17-2.40]	*0.51*			
Unknown	44 (15.7)	23	0.74	[0.21-2.62]	*0.64*			

^£^ n = 241 visits for 152 patients; 3 visits were removed after the residual analysis.

^§^ in years.

* During the previous 4 weeks.

** Beck ≥ 9.

*** CES-D score >17 for males and >23 for females.

‡ AUDIT score ≥13.

The results of the multivariate analysis are presented in Table 3. Five variables remained associated with reporting at least one HCV risk practice: younger age, suicidal risk, cocaine use, alcohol dependence and living in a couple. Compared with patients who were living alone, the increasing risk found in patients living in a couple in the univariate analysis was confirmed after multiple adjustment, i.e. an increased risk of HCV risk practices was observed among individuals living in a couple with a non-drug user and in those living in a couple with a drug user. Individuals living with a drug user but not in a couple exhibited similar risks to those of participants who lived alone. HCV status was not found to be associated with the outcome.

When performing the sensitivity analysis excluding the sharing of toiletry items, two variables remained associated with reporting at least one HCV risk practice: suicidal risk (IRR[CI95%] = 6.00 [1.80-20.02]) and living in a couple with a drug user (IRR[CI95%] = 6.72 [1.82-24.80]).

## Discussion

The findings of our study are important to better understand factors associated with HCV risk practices in methadone maintained opioid-dependent individuals.

Although the literature shows that methadone maintenance treatment has a positive impact on HCV transmission risk reduction [20,21], it is also known that methadone treatment is not enough to sufficiently reduce the HCV transmission risk in opioid-dependent individuals [22]. Indeed, in this study conducted on this population, it appears that duration on MMT does not have a significant impact on HCV risk practices. However, considering HCV prevalence in the whole population of drug users, and also the high percentage of those unaware of their HCV status (15% in our sample but much higher in other studies (e.g. 65% in [23])), the impact of HCV risk practices is a crucial point to address in this population. These findings confirm the need for combined approaches including access to needle and syringe programs and opioid maintenance treatment [22,24].

When investigating correlates of HCV risk practices, several factors remain associated in the final model. First, people who were younger were more likely to report such practices. A similar result was found elsewhere [25]. In addition, Fuller et al. showed that among PWID, HCV seroconverters tended to be younger [26], while another study found that PWID with high-risk profiles were younger [27,28]. This fact underlines the importance of targeting younger drug users using specific interventions [29].

In our study, cocaine use was associated with HCV risk practices. This finding reflects existing literature which shows that cocaine and crack use are recurrent correlates of HCV seropositivity [30,31]. It is known that the more frequent the drug intake in cocaine users the more they engage in risk practices [32-34]. Alcohol use is also a well-known factor of HCV and HIV risk practices in opioid-dependent individuals on MMT [35]. In addition, alcohol is also associated in PWID with unsafe sexual and injecting practices [36]. Results from some studies have suggested the effectiveness of implementing a “motivational-based” intervention in order to reduce alcohol use among HCV-infected individuals [37,38].

Our findings on HCV risk practices showed an association with suicidal risk. Indeed, many studies investigating factors linked to attempted suicide, which is intimately correlated with suicidal risk [39], have found several associated risk practices, such as non-suicidal self-harm incidents [40], risky health practices [41] and overdoses [42]. Moreover, it has been shown that PWID with suicidal ideation reported receiving and providing used syringes more frequently [43].

Finally, methadone-maintained patients living in a couple were more likely to report HCV risk practices. This result is important as it provides the opportunity to identify prevention strategies. More particularly, those living in a couple with a non-drug using partner and a drug-using partner, respectively, were four and ten times more likely to report HCV risk practices than those who were living alone. This at-risk group deserves special attention. A common source of HCV infection in both members of a couple has been already demonstrated, supporting the hypothesis that HCV transmission is frequent within couples [44] or at least in an intra-familial context [10]. In some situations, “living in a couple” may translate into a greater probability of knowing the serostatus of one’s partner. In turn, this knowledge may have an important impact on whether an individual engages in either protective or indeed so-called “risk” practices. However, in a study on a prevention intervention for couples where one or both partners were PWID, El-Bassel et al. [45] showed a high prevalence of HIV and HCV, respectively 28% and 75%, and that a quarter of those who tested positive in that study were unaware of their status. Ignorance about one’s own status is a phenomenon which deserves greater consideration and investigation. It is not surprising that couples share toiletry items more than people living alone. However, because HCV infection is very prevalent in drug users, it would seem important to pay attention to this risk practice in drug-using couples. A recent meta-analysis showed that the risk of HCV infection through shared drug preparation equipment was similar to that of shared syringes and that the infection status of the sharing partner was often unknown [46]. Moreover, it has been shown that in regular heterosexual relationships, injecting practices are not organized around HCV status but are more influenced by the couple’s connection with other drug users and more particularly whether they share equipment with those outside the couple more frequently [47]. In other words, the more HCV risk practices they are involved in outside the couple, the more they share equipment with their partner. All these results should be taken into account to target methadone-maintained patients, especially when their partner is a drug user, and to individually adapt prevention counselling to reduce the risk of HCV transmission. One previous article has already pointed out that health education materials for HCV prevention pay insufficient attention to couples [48]. For example, transition to injection within intimate partnerships is known to be common. Couples-focused interventions should concentrate more on this issue [49]. As has already been demonstrated for HIV prevention regarding sexual risk practices in couples, there is a need to focus on how PWID couples manage HCV risk transmission, without perpetuating risk equivalence beliefs between HIV and HCV transmission [50]. More generally, as suggested in a recent study, methadone maintenance treatment complemented with psychosocial interventions can help to reduce HCV risk practices [51].

The absence of any association between HCV seropositivity and HCV risk practices has to be discussed. Indeed, it shows that those who are HCV positive are not less likely to engage in HCV risk practices. This confirms the importance of setting up individually (or couple-based) adapted prevention interventions to reduce the risk of HCV transmission. Moreover, 15% of individuals were not aware of their HCV status. This result should encourage the promotion of HCV testing in the drug using population and more particularly in methadone-maintained patients.

Some limitations have to be acknowledged. First, as we excluded triple co-dependent individuals from the trial, it is possible that most-at-risk individuals were excluded. Furthermore, evaluating changes in risk practices over just one year in this small sample with a low prevalence of risk practices at baseline is difficult. Data were for the most part self-reported and accordingly social desirability bias is possible. Nevertheless, the reliability of self-reports in drug-using populations has already been demonstrated [52]. Finally, one important limitation is that we included non-drug related practices in the factors’ analysis yet it is well known that these practices are not as important in HCV transmission as drug related risk practices (including syringe- and paraphernalia-sharing).

## Conclusions

Although the HCV epidemic is a growing health issue in increasingly more countries, especially in drug-using populations, current access to care and to prevention interventions is not sufficient.

Identifying individuals at risk of HCV transmission during methadone treatment, such as stimulant users, alcohol dependent individuals, and those at suicidal risk, is necessary to optimize response to treatment. Innovative prevention approaches tailored to couples are urgently needed and could decrease the risk of HCV in this population.

## Abbreviations

ANRS: Agence Nationale de Recherche sur le Sida et les Hépatites; ANSM: Agence Nationale de Sécurité du Médicament; AUDIT: Alcohol use disorders test; BHS: Beck hopelessness scale; BBV-TRAQ: Blood-borne virus transmission risk assessment questionnaire; CATI: Computer-assisted telephone interview; CEIP: Center of evaluation and information on drug dependence; CES-D: Center for epidemiological study depression; CI: Confidence interval; HCV: Hepatitis C virus; HIV: Human immunodeficiency virus; INSERM: Institut National de la Santé et de la Recherche Médicale; IQR: Interquartile range; IRD: Institut de Recherche pour le Développement; M0: M3, M6, M9, Month 0, month 3, month 6, month 9; MMT: Methadone maintenance treatment; OR: Odds ratio; ORS PACA: Observatoire Régionale de la Santé Provence-Alpes-Côte d’Azur; OTI: Opiate treatment index; PWID: People who inject drugs; UMR: Unité Mixte de Recherche.

## Competing interests

The Authors taking part in this study declare they have nothing to disclose regarding funding or competing interest with respect to this manuscript.

## Authors’ contributions

MPC, LM, PR, BS, JPD, AM and JCD designed the study and wrote the protocol. PR and MPC managed the literature searches, formulated the research questions and PR wrote the first draft of the manuscript. CL, FM and MM participated in the data collection and CL and FM undertook the statistical analyses. All authors contributed to and have approved the final manuscript.
